# Insights into the potential mechanism of Beiqishen Jiangtang Granule in the treatment of type 2 diabetes nephropathy: A study based on network pharmacology, molecular docking, and biological validation

**DOI:** 10.1097/MD.0000000000047533

**Published:** 2026-02-13

**Authors:** Meitong Pan, Panpan Wang, Xinxin Wang, Zhen Wang, Zhanping Zhang, Keke Yang, Weili Liu, Wei Ma, Xiubo Liu

**Affiliations:** a Department of Pharmacy, Heilongjiang University of Chinese Medicine, Harbin, China; b Key Laboratory of Xinjiang Phytomedicine Resource and Utilization, Ministry of Education, Department of Pharmacy, Shihezi University, Shihezi, China; c Department of Jiamusi, Heilongjiang University of Chinese Medicine, Jiamusi, China.

**Keywords:** Beiqishen Jiangtang Granule, diabetes mellitus, gut microbiota, mechanism of action, network analysis

## Abstract

Beiqishen Jiangtang Granule, derived from Huangqi Liu Yi Tang and Shengmai Yin, plays a significant role for the therapy of type 2 diabetes mellitus (T2DM). However, the underlying molecular therapeutic mechanisms remain unclear. This study uses an integrated approach combining network pharmacology, molecular docking, and in vitro validation experiments to explore potential bioactive compounds, key targets, major signaling pathways, and underlying molecular mechanisms of Beiqishen Jiangtang Granule in the treatment of T2DM. In this study, network analysis was employed to screen metabolites and potential targets using the TCMSP database, followed by Gene Ontology, kyoto encyclopedia of genes and genomes enrichment analyses to predict the underlying mechanisms, and the protein encoded by the core target was docked with the active ingredient. Using a streptozotocin-induced approach, a T2DM rat model was established to evaluate the bioactivity of Beiqishen Jiangtang Granule on blood glucose levels, lipid profiles, inflammatory cytokines, gene expression, and gut microbiota composition. Network analysis identified 121 primary metabolites and 30 key targets in Beiqishen Jiangtang Granule. Integrated Gene Ontology and kyoto encyclopedia of genes and genomes analyses (27 items) predicted that Beiqishen Jiangtang Granule regulates serum contents of insulin, tumor necrosis factor-alpha, and interleukin-6, and modulates TP53, Akt, and PI3K protein expression to exert hypoglycemic effects. Animal studies confirmed Beiqishen Jiangtang Granule significant glucose-lowering action via these pathways and mRNA regulation. Additionally, Beiqishen Jiangtang Granule was shown to rebalance gut microbiota by enriching beneficial bacterial communities and suppressing the growth of harmful bacteria, aiding T2DM treatment. These findings demonstrate Beiqishen Jiangtang Granule’s characterized by multiple components, targets, and pathways mechanisms in treating T2DM. Beiqishen Jiangtang Granule have a very significant therapeutic effect on T2DM by regulating the concentrations of insulin, tumor necrosis factor-alpha and interleukin-6 in serum and influencing the expressions of TP53, Akt and PI3K proteins and mRNA. In addition, Beiqishen Jiangtang Granule can also regulate the imbalance of intestinal flora related to T2DM in patients by promoting the proliferation of beneficial bacteria and inhibiting harmful bacteria, thereby assisting T2DM.

## 1. Introduction

Diabetes mellitus (DM) is a chronic metabolic disease with insulin (INS) resistance (pathological basis) and impaired pancreatic β-cell function.^[1]^ INS resistance reduces INS sensitivity and induces hyperinsulinemia, while β-cell dysfunction impairs INS secretion, collectively disrupting glucose homeostasis.^[2]^ Type 2 diabetes mellitus (T2DM) accounts for over 90% of DM cases,^[3]^ with its global incidence rising and threatening human health. T2DM is often asymptomatic initially but progresses to classic “3 polys and 1 weight loss” (hyperglycemia-mediated polyuria, polydipsia, polyphagia, weight loss).^[4]^ Advanced DM brings acute/chronic complications: metabolic disorder-induced damage to eyes, kidneys, liver, cardiovascular/nervous systems, and recurrent skin infections.^[5-8]^ T2DM treatment relies on lifestyle modifications.^[9-11]^ When insufficient, interventions include oral hypoglycemic agents, INS therapy, surgery, and other adjuvants.^[12]^

Most traditional Chinese medicines (TCMs) are natural drugs containing various chemical components. When combined in T2DM treatment, Chinese herbal medicines highlight a holistic intervention strategy.^[13,14]^ In addition, TCM compound prescriptions can provide additional benefits through comprehensive management and regulation of intestinal flora, offering important adjuvant treatment for T2DM. This approach effectively controls T2DM, prevents complications, and improves the quality of life for patients. While modern Western medicine clinical practice is optimizing the treatment methods for diabetes, in the face of the constantly growing group of diabetes patients, its treatment methods are facing enormous challenges and gradually exposing their corresponding side effects.^[15]^ At this point, the unique advantages of TCM in treating T2DM, such as high stability of metabolic products, low toxicity, few side effects, and convenient use, have emerged.^[16,17]^ Guided by the concept of holistic regulation, TCM demonstrates unique advantages and application potential in preventing and treating diabetes and its complications.

Beiqishen Jiangtang Granule, a functional food, integrates the formulation concepts of Huangqi Liuyi Tang from Taiping Huimin Heji Jufang and Shengmai Yin from Nei Wai Shang Bian Huo Lun.^[18,19]^ Based on extensive literature references and repeated deliberations by renowned experts, the formula of Beiqishen Jiangtang Granule was finally determined as: *Panax ginseng* 2 g, *Astragalus membranaceus* 30 g, *Schisandra chinensis* 3 g, and *Glycyrrhiza uralensis* 5 g. Beiqishen Jiangtang Granule is a key method for the clinical treatment of T2DM, offering advantages through its multiple components, pathways, and targets. However, this feature is also the biggest obstacle for us to explain the fundamental cause and mechanism by which it treats type 2 diabetes. Network pharmacology, as a research method that combines bioinformatics, molecular biology, and multiple databases. The mechanism of action of multicomponent drugs can be consulted and screened. Additionally, the therapeutic mechanisms of these metabolites were systematically studied at both systemic and molecular levels using the T2DM rat model. The research results not only provide a solid theoretical and experimental foundation for treating T2DM with Beiqishen Jiangtang Granule, exploring key metabolites and metabolic pathways, but also broaden the application exploration of TCM in diabetes prevention and management. These insights are highly valuable for guiding future drug development

## 2. Material and methods

### 2.1. Drugs and reagents

The 4 herbs that make up the Beiqishen Jiangtang Granule: *P ginseng*, *A membranaceus*, *S chinensis*, and *G uralensis*. The First Affiliated Hospital of Heilongjiang University of Chinese Medicine, China, is the provider of all materials. The reagents and consumables used in the experiments included: blood glucose test strips and single-use lancets for capillary blood collection from Yuyue Medical Equipment Co., Ltd. (Jiangsu, China); streptozotocin (STZ) from Coolab Technology Co., Ltd. (Beijing, China); in this study, metformin hydrochloride tablets were produced by Merck Pharmaceutical Co., Ltd. (Jiangsu, China), while citric acid and sodium citrate dihydrate were supplied by Sinopharm Chemical Reagent Co., Ltd. (Shanghai, China); disposable venous blood collection needles from Yongkang Medical Products Co., Ltd. (Shandong, China); disposable negative-pressure blood collection tubes from Aosait Medical Technology Co., Ltd. (Shandong, China); edible glucose, Bradford protein concentration assay kit, SDS-PAGE gel preparation kit, and ECL chemiluminescent detection kit from Beyotime Biotechnology Co., Ltd. (Shanhai, China); phenylmethylsulfonyl fluoride from Amresco (USA); substantial RIPA lysis buffer, 5× protein loading buffer, 10× TBS buffer, 10× SDS-PAGE protein electrophoresis buffer, skimmed milk powder, and protein marker from Lankerc Technology Co., Ltd. (Beijing, China); PVDF membrane from Lankerc Technology Co., Ltd. (Beijing, China); thick transfer blotting paper from Bio-Rad Life Science Products Co., Ltd. (Shanghai, China); β-actin, Rabbit Anti-TP53 (wt-p53) antibody, rabbit anti-AKT1 antibody, rabbit anti-PI3 Kinase p110 β antibody. Reagents used in this study included: goat anti-rabbit IgG H&L/HRP antibody from Bioss Biotechnology Co., Ltd. (Beijing, China); blood/cell/tissue genomic DNA extraction kit, genomic DNA extraction kit, and universal DNA purification & recovery kit from Tiangen Biotech Co., Ltd. (Beijing, China); rat INS, tumor necrosis factor-alpha (TNF-α), and IL-6 ELISA Kits from Jingmei Biotechnology Co., Ltd. (Jiangsu, China); Bradford Protein Concentration Measurement Kit from Biyuntian Biotechnology Co., Ltd.; and SDS-PAGE gel Preparation Kit from Lanjieke Technology Co., Ltd. (Beijing, China); and ECL Chemiluminescence Detection Kit of Meilun Biotechnology Co., Ltd. (Dalian, China).

### 2.2. Instruments and equipment

The equipment used in this study includes a glucometer from Yuyue Medical Equipment Co., Ltd. (Jiangsu, China), microplate reader from Diatek Corporation (West Bengal, India), medical centrifuge from Xiangyi Centrifuge Instrument Co., Ltd. (Changsha, China), glass homogenizer from Hua Heng Biotechnology Co., Ltd. (Nantong, China), Trans-SD universal semidry transfer electrophoresis tank Kaiyuan Xinerui Instrument Co., Ltd. (Beijing, China), GE AI600 series ultra-sensitive multifunctional imager from Dequan Xingye Trading Co., Ltd. (Beijing, China), quantitative PCR from Agilent Technologies Co., Ltd. (Beijing, China), and XINDNA multi-purpose horizontal electrophoresis tank from Qinxiang Scientific Instrument Co., Ltd. (Shanghai, China). Blood glucose meter (Jiangsu Yuyue Medical Equipment Co., Ltd., Jiangsu, China), microplate reader (Diatek Company, Wuxi, China), trans-SD universal semidry transfer electrophoresis tank (Beijing Kaiyuan Xinrui Instrument Co., Ltd., Beijing, China), American GEAI600 series ultra-sensitive multifunctional imager (Beijing Dequan Xingye Trading Co., Ltd., Beijing, China) Fluorescence quantitative PCR (Agilent [China] Technology Co., Ltd.).

### 2.3. Preparation of Beiqishen Jiangtang Granule

The production process of Beiqishen Jiangtang Granule is as follows: Soak 20 g of *P ginseng*, 300 g of *A membranaceus*, 30 g of *S chinensis*, and 50 g of *G uralensis* in water for 1.5 hours with a solid-to-liquid ratio of 1:20, and the number of extractions is 2 times under these extraction conditions. Then, combine and concentrate the extracts and prepare them by freeze-drying. Start the heating plate and set it to 40°C, and increase the temperature by 10°C every 10 minutes until it reaches 70°C. After the moisture content curve stabilizes, maintain it for 30 minutes to complete the sublimation drying. Finally, raise the temperature to 90°C, decrease the vacuum degree from 23 to 25 Pa, and perform desorption drying after the moisture content curve stabilizes. The specification of the punching machine is 15 g/bag.

### 2.4. Network analysis prediction

#### 2.4.1. Main metabolites of Beiqishen Jiangtang Granule

In this study, the Traditional Chinese Medicine Systems Pharmacology Database and Analysis Platform (TCMSP, accessible at https://www.tcmsp-e.com/load_intro.php?id=43) served to identify key metabolites (n = 109) in Beiqishen Jiangtang Granule. *P ginseng*, *A membranaceus*, *S chinensis*, and *G uralensis* were used as search terms. Metabolites underwent screening based on thresholds of drug-likeness (DL ≥ 0.18) and oral bioavailability (OB ≥ 30%), after which potential targets were predicted. Target annotations were normalized via the UniProt database (https://www.uniprot.org/), and the metabolite-target regulatory network was built using Cytoscape 3.8.0 software (Cytoscape Consortium, Seattle) to clarify their intricate interactions.

#### 2.4.2. Screening of targets related to DM

The GeneCards (https://www.genecards.org/), OMIM (https://omim.org/), and DRUGBANK (https://www.drugbank.ca/) databases were used in this study, we retrieved data using “Diabetes Mellitus” as keywords. After integrating the data, we removed duplicate values to determine the targets for diabetes DM. Conversion of protein names to gene names was performed using the UniProt database. Finally, we visualized the results using the Microbial Informatics and Genomics website (http://www.bioinformatics.com.cn).

#### 2.4.3. Construction of interaction network between metabolites of Beiqishen Jiangtang Granule and diabetic target proteins

The Venny 2.1.0 tool (https://bioinfogp.cnb.csic.es/tools/venny), the intersection of targets associated with Beiqishen Jiangtang Granule metabolites and DM targets was identified and visualized with a Venn diagram. Intersecting targets were submitted to the STRING database (the confidence levels are high, medium, and low, respectively: 0.9, 0.7, and 0.4; https://string-db.org/) for constructing a protein–protein interaction (PPI; threshold 0.7) network model. Finally, topological analysis was carried out via Cytoscape 3.8.0 software (https://cytoscape.org/) to identify key targets for treating DM.

#### 2.4.4. GO enrichment and KEGG pathway enrichment analysis

The 3 categories of Gene Ontology (GO), namely molecular function (MF), biological process (BP), and cellular component (CC), constitute the core of its annotation framework. By entering 199 drug-disease intersecting targets into the Metascape platform, functional enrichment analysis for GO can be efficiently executed.^[20]^

In this study, enrichment analysis of kyoto encyclopedia of genes and genomes (KEGG) pathways was conducted via the Metascape platform (accessible at https://metascape.org/gp/index.html#main/step1), and the results were visualized using the Microbial Informatics and Genomics website. Subsequently, the relationships between the metabolites of Beiqishen Jiangtang Granule, DM targets, and their action pathways were constructed via Cytoscape 3.8.0 software. Topological network analysis was carried out for identifying major metabolites and key targets.

#### 2.4.5. Molecular docking

In this study, 5 active compounds (quercetin, kaempferol, formononetin, isomucronulatol 7-*O*-glucoside, and naringenin) with the highest degree values in the compound-target-pathway network were selected for molecular docking against 5 core target proteins (interleukin-6 [IL-6], TP53, INS, TNF, and AKT1) identified from the protein–protein interaction (PPI) network; specifically, the compounds were first imported into Chem3D software for energy minimization using the MM2 force field, and the processed structures were saved in MOL2 format for subsequent analysis. Validated human protein sequences corresponding to the core targets were retrieved from the UniProt database, and their 3-dimensional (3D) structures were downloaded from the Protein Data Bank database; the retrieved protein structures were then preprocessed using PyMOL software, which involved removing water molecules, repairing and optimizing side chains, and adding hydrogen atoms. After preprocessing, the target proteins were further handled with AutoDock software, molecular docking simulations were performed using AutoDock Vina, and the binding modes between compounds and target proteins were visualized using PyMOL software.

### 2.5. In vivo experimental verification

#### 2.5.1. Establishment of T2DM rat model

Sixty male SPF-grade SD rats (200 ± 20 g body weight) utilized in the experiment were procured from Changsheng Biotech Co., Ltd. (Liaoning, China) and acclimatized for 1 week at a stable temperature of 24 ± 2°C. Eight rats were randomly chosen as thenormal control cohort, and the leftover 52 rats were used for T2DM model establishment via high-fat diet combined with STZ induction.

Based on the adult dosage of 40 g/d, and by references and the body surface area ratio between animals and humans, the low-, medium-, and high-dose regimens for rats were computed as 1.35, 2.7, and 5.4 g/kg, in that order. After formulating this preparation, the adult dosage was set at 1 bag/d (15 g/d), with the corresponding low-, medium-, and high-dose regimens for rats adjusted to 0.51, 1.02, and 2.04 g/kg, sequentially.

Starting from week 2, rats in the model group were subjected to a 4-week high-fat diet protocol. During this period, body weight, fasting blood glucose were measured, and food and water consumption were calculated weekly. After 4 weeks, model group rats received an intraperitoneal STZ injection at 40 mg/kg, while the normal group received a citrate-citrate buffer solution. Three days later, fasting blood glucose was measured after a 12-hour fast, and again 2 days later. Rats with lower blood glucose levels were given a supplementary injection of STZ at 15 mg/kg. Finally, 48 rats exhibiting fasting blood glucose ≥ 16.7 mmol/L and presenting symptoms such as polydipsia, polyphagia, polyuria and weight loss were chosen as experimental subjects for the model.

#### 2.5.2. Animal grouping and treatment

Based on body weight, rats were randomly assigned to 5 groups: normal, model control, Beiqishen Jiangtang Granule low/medium/high-dose, and metformin hydrochloride, with 8 rats in each group. Physiological saline was injected into the normal and model control groups, while the metformin hydrochloride group received 200 mg/kg metformin hydrochloride. The remaining groups were treated daily with their respective doses of Beiqishen Jiangtang Granule for 4 weeks. After the treatment period, all rats underwent a 12-hour fast, were anesthetized with sodium pentobarbital, and had blood samples collected from the abdominal aorta, and blood samples were collected from the abdominal aorta. Liver and kidney tissues were also harvested for further analysis.

### 2.6. Sampling and STZ-induced T2DM rat index measurements

#### 2.6.1. Taking materials

After anesthetizing rats with sodium pentobarbital, blood was sampled from the abdominal aorta and allowed to coagulate at room temperature for 1 hour. Subsequent to coagulation, samples underwent centrifugation at 3000 rpm for 20 minutes at 4°C to isolate serum, which was preserved at −80°C. Liver and kidney tissues were dissected, rinsed with physiological saline, blotted dry using filter paper, and weighed to calculate organ indices. Additionally, fresh cecal content samples (≥0.2 g) were gathered and cryopreserved at −80°C for follow-up analyses.

#### 2.6.2. Monitoring of rat physiological parameters

During the experiment, daily records of water and food intake were maintained for all rats. Furthermore, rats’ body weight was assessed weekly before feeding.

#### 2.6.3. Fasting blood glucose levels and an oral glucose tolerance assessment

Following a 12-hour fasting period, fasting blood glucose values were measured once weekly using the method of tail vein blood collection. Following the fast, rats were gavaged with the corresponding drugs. Half an hour later, a 25% glucose solution (2 g/kg) was given orally based on the body weight of the rats. Tail vein blood samples were acquired at 0, 30, 60, 90, and 120 minutes after glucose administration to measure blood glucose levels. Subsequently, the area under the curve (AUC) was computed to evaluate glucose tolerance status.

#### 2.6.4. Determination of biochemical indicators using automated biochemical analyzer

An automated biochemical analyzer was utilized for quantifying levels of triglycerides (TG), total cholesterol (CHOL), high-density lipoprotein cholesterol (HDL-C), low-density lipoprotein cholesterol (LDL-C), alanine aminotransferase (ALT), and aspartate aminotransferase (AST).

#### 2.6.5. Determination of ELISA indicators

The concentrations of INS, tumor necrosis TNF-α, and IL-6 were quantified via enzyme-linked immunosorbent assay (ELISA). Optical density readings were obtained at a 450 nm wavelength per the kit’s instructions, and the homeostasis model assessment for insulin resistance (HOMA-IR) was calculated.

#### 2.6.6. Determination of TP53, PI3K, and Akt mRNA expression in liver tissue by qRT-PCR

Total RNA was extracted from rat liver tissue, and cDNA was generated following the kit instructions, followed by qRT-PCR analysis. Primers were constructed by Beijing Ruiboxingke Biotechnology Co., Ltd., with sequences listed in Table S1, Supplemental Digital Content, https://links.lww.com/MD/R301. Each sample was analyzed in triplicate, and relative gene expression was calculated via the 2^−ΔΔCt^ method.^[21]^

#### 2.6.7. Western-blot determination of TP53, PI3K, Akt mRNA expression in liver tissue

Approximately 50 mg of rat liver tissue was added to 500 μL of lysis buffer containing 5 μL of PMSF, then homogenized on ice. The homogenate was kept on ice for 1 hour, followed by centrifugation at 14,000 rpm for 20 minutes at 4°C. The supernatant was harvested, and protein concentration was assessed by the BCA method. Samples were mixed with loading buffer and heated at 95°C for 5 minutes for protein denaturation, then separated via SDS-PAGE and transferred onto a PVDF membrane. The membrane was blocked with 5% skim milk in TBST for 2 hours at room temperature, rinsed 3 times with TBST, and incubated with primary antibodies overnight at 4°C. After washing, HRP-conjugated secondary antibodies were added for 1 hour at room temperature. Following final rinses, the membrane was processed with ECL substrate, and band intensities were evaluated using ImageJ software. Antibody dilutions were as follows: primary antibodies at 1:1000; secondary antibodies at 1:2000; AKT at 1:2000; PI3Kβ at 1:1000.

### 2.7. Statistical analysis

Data are presented as mean ± standard deviation (*x* ± *s*). Analytical evaluations, including Shapiro–Wilk normality testing and Levene’s test for variance homogeneity, were conducted with GraphPad Prism 8.0 software. When these assumptions were satisfied, 1-way analysis of variance (ANOVA) was utilized; otherwise, the Kruskal–Wallis test was employed. A *P*-value < .05 was deemed to denote a statistically significant difference (each group conducted 3 repetitions of the experiment).

### 2.8. Analysis of rat intestinal flora

#### 2.8.1. Total DNA extraction and sample detection

Total DNA was isolated from fecal samples of 6 rat groups (six samples per group) using the TianGen DNA extraction kit. DNA purity and concentration were evaluated via 1% agarose gel electrophoresis, and samples were subsequently diluted to 1 ng/μL.

#### 2.8.2. Amplification of PCR products

PCR amplification was carried out using 16S rRNA V3-V4 region primers (341F: 5′-CCTAYGGGRBGCASCAG-3′; 806R: 5′-GGACTACNNGGGTATCTAAT-3′). The reaction mixture consisted of 15 µL of Phusion^®^ High-Fidelity PCR Master Mix, 0.2 µM primers, and 10 ng of template DNA. Amplification conditions were as follows: initial denaturation at 98°C for 1 minute, 30 cycles of 98°C for 10 seconds, 50°C for 30 seconds, and 72°C for 30 seconds, with a final extension at 72°C for 5 minutes.

#### 2.8.3. Pooling and purification of PCR products

PCR products underwent 2% agarose gel electrophoresis. Eligible samples were purified via magnetic beads, quantified, pooled at uniform concentrations, and reanalyzed by 2% agarose gel electrophoresis. Finally, target bands were retrieved using the TianGen Universal DNA Purification and Recovery Kit.

#### 2.8.4. Library construction and sequencing

Libraries were constructed with the NEB Next^®^ Ultra™ II FS DNA PCR-free Library Preparation Kit. After quantification via Qubit and qRT-PCR, sequencing was performed on the NovaSeq6000 platform with PE250 sequencing (each group conducted 3 repetitions of the experiment).

## 3. Results

### 3.1. Metabolites and related targets of Beiqishen Jiangtang Granule

**Figure 1.**
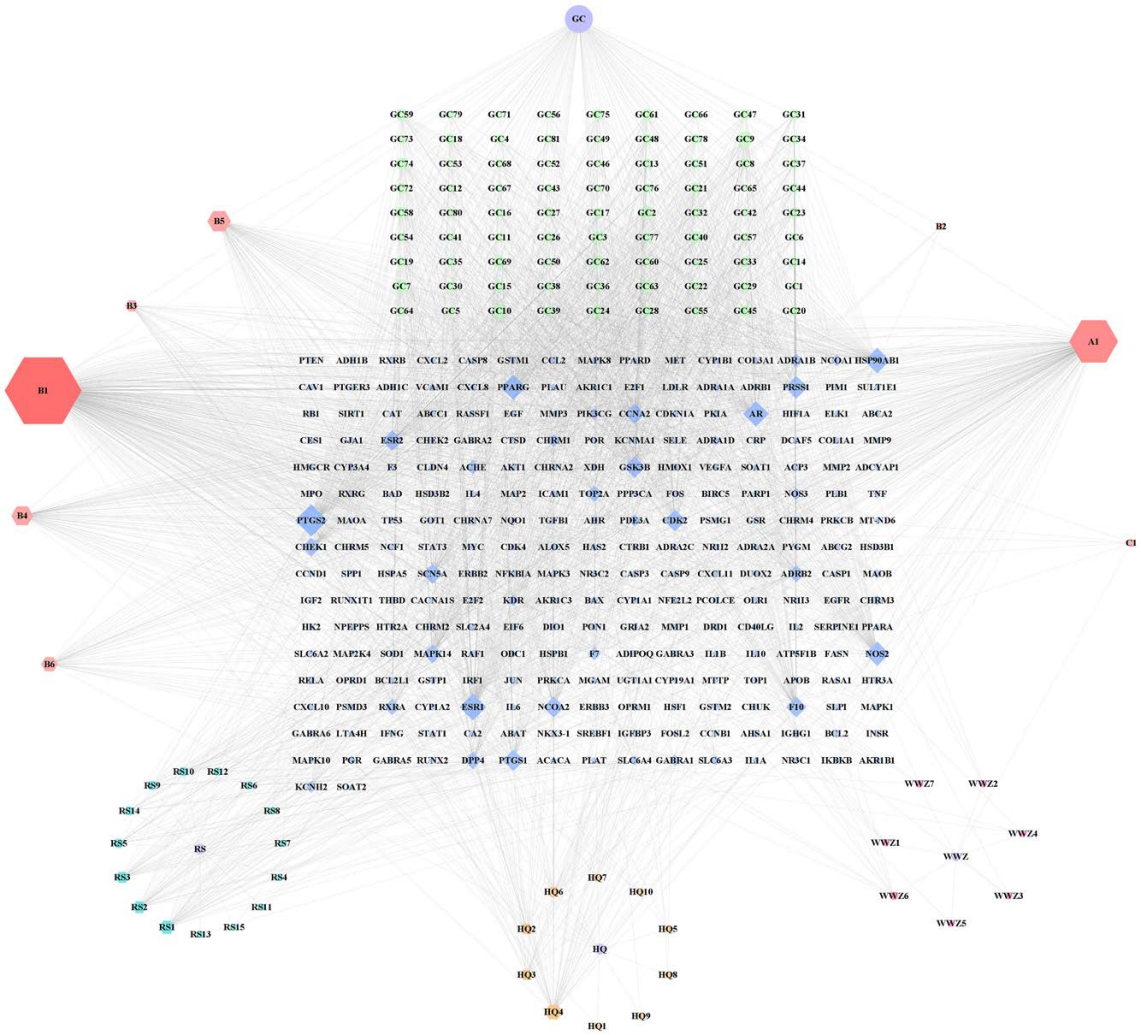
Network diagram of drugs and main metabolites and targets. Blue circular nodes represent drugs, hexagonal nodes represent metabolites, the metabolites of different drugs are represented by different colors, and the targets are represented by blue diamond nodes.

Using the TCMSP database, we identified 121 major metabolites of Beiqishen Jiangtang Granules using screening criteria of oral bioavailability ≥ 30% and drug-likeness ≥ 0.18. These metabolites included quercetin, kaempferol, and formononetin, among others. Moreover, 30 key targets including AKT1, TNF-α, IL6, TP53, VEGFA, and CASP3 were successfully identified. The drug-component-target relationships were visualized using Cytoscape 3.8.0 software, resulting in a network comprising 367 nodes and 2370 edges. This network comprehensively illustrates the multicomponent and multi-target properties possessed by Beiqishen Jiangtang Granule (Fig. 1, Table S2,Supplemental Digital Content, https://links.lww.com/MD/R301).

### 3.2. Acquisition of DM-related targets

Four databases were used to retrieve DM-related targets: OMIM, DRUGBANK, DisGeNET, and GeneCards. The number of targets obtained from each database was as follows: 257 from OMIM, 11 from DRUGBANK, 3134 from DisGeNET, and 3454 from GeneCards. After merging the data and removing duplicates, a total of 5039 DM-related targets were identified.

### 3.3. Protein interaction network construction

**Figure 2.**
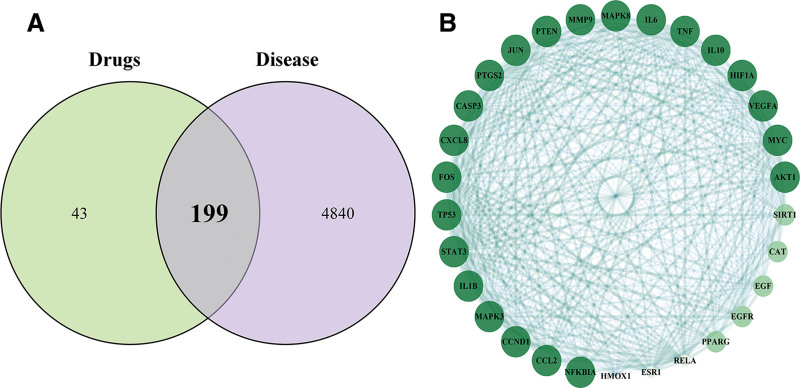
(A) Venn diagram of drugs and disease targets. (B) Core PPI network. PPI = protein–protein interaction.

A total of 199 intersections of the ingredient targets of Beiqishen Jiangtang Granule with DM targets were presented in a Venn diagram (Fig. 2A). Analysis of these targets was performed using the STRING platform, and using Cytoscape 3.8.0 software, a network diagram of potential target protein interactions was constructed. The average degree value (DC) for this network was calculated, nodes with degrees in the interval above twice the average degree were defined as core target proteins (Fig. 2B). In the graph, node size and color intensity were used to visualize degree values, while the color intensity and thickness of the connecting lines indicate the interaction strength. Thirty targets were screened, of which 22 proteins had a degree value of 29, including AKT1, NFKBIA, CCL2, CCND1, MAPK3, IL1B, STAT3, TP53, FOS, CXCL8, CASP3, PTGS2, JUN, PTEN, MMP9, MAPK8, IL-6, TNF-α, IL10, HIF1A, VEGFA, and MYC; 5 proteins had a degree value of 28, 2 had a degree value of 27, and 1 had a degree value of 26. As the degree value increases, the broader the relevance of the target and the stronger the effect, indicating that Beiqishen Jiangtang Granule may primarily exert their therapeutic effects on DM through these targets.

### 3.4. GO enrichment, signaling pathway enrichment analysis using the KEGG database, and molecular docking

**Figure 3.**
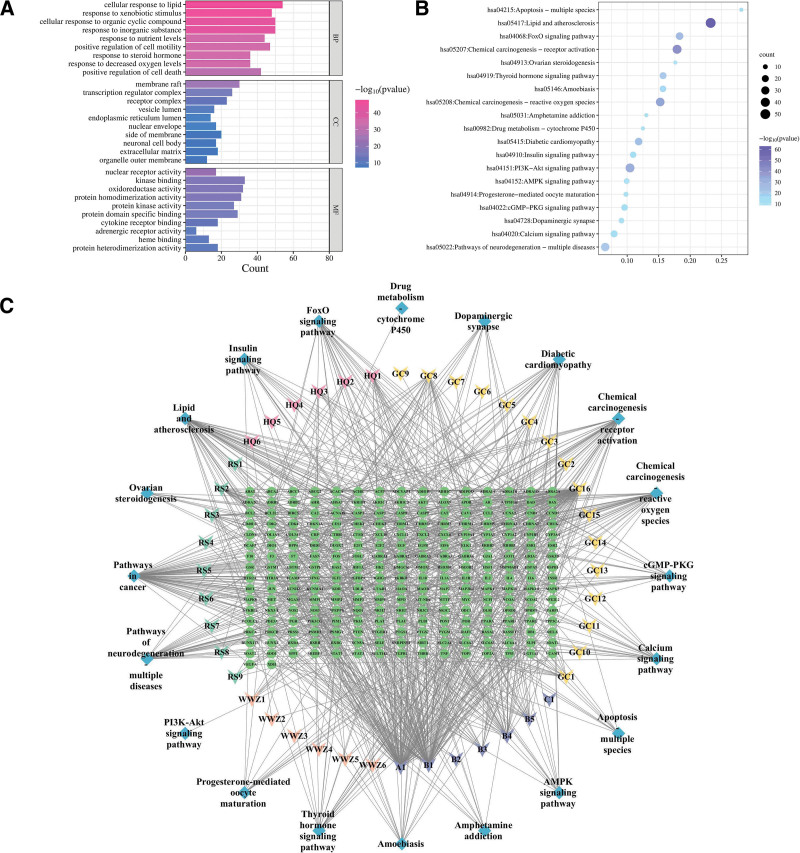
(A) GO enrichment analysis of potential targets for major metabolites in Beiqishen. (B) KEGG pathways of anti-FC targets in Beiqishen. (C) Network mapping active components–targets–pathways of Beiqishen Jiangtang Granule. GO = Gene Ontology, KEGG = kyoto encyclopedia of genes and genomes.

The potential targets of Beiqishen Jiangtang Granule for treating DM were analyzed using the Metascape platform for GO enrichment analysis, focusing on BP, CC, and MF. The results were sorted by ascending *P*-value, and the top 10 terms for each category were selected and visualized using the MicroSignal platform (Fig. 3A). The GO enrichment analysis revealed that the BP terms associated with Beiqishen Jiangtang Granule included cellular responses to lipid, organic cyclic metabolites, and inorganic substances. The CC terms were primarily associated with membrane raft and transcription regulator complex, while the MF terms were enriched in kinase binding, oxidoreductase activity, and protein homodimerization activity.

In the enrichment analysis, 215 KEGG pathways were confirmed in this study. To better present the results, the *P*-values were sorted in ascending order, and subsequently, the top 20 pathways were selected for visualization using the MicroSignal platform (Fig. 3B). In the figure, the color differences of the bubbles indicate varying enrichment levels, while the size reflects the quantitative count of enriched genes. The main pathways involved include the lipid metabolism-atherosclerosis axis, PI3K-AKT/FoxO signaling axis, and INS signaling pathway related to diabetic complications. These results suggest that the development and progression of DM involve multiple factors, and Beiqishen Jiangtang Granule may exert their therapeutic effects on DM by modulating multiple metabolic pathways.

Based on pathway and related target information, the Cytoscape 3.8.1 software (GraphPad Software, Inc., San Diego) was used to construct a network of components of Beiqishen Jiangtang Granule, DM targets, and related pathways (Fig. 3C). This network contained 306 nodes and 971 edges. Forty-four inverted triangles denoted active components of Beiqishen Jiangtang Granule (excluding those lacking target information in TCMSP), with distinct colors for different drug components: pink for *A membranaceus*, yellow for *G uralensis*, orange for *S chinensis*, cyan for *P ginseng*, and purple for shared components. A1 was common to *P ginseng*, *A membranaceus*, and *G uralensis*; B1, B2, B3, and B4 were shared by *A membranaceus* and *S chinensis*; C1 was common to *P ginseng* and *S chinensis*. Twenty blue thrombus nodes denoted core pathways, and 242 green circular nodes represented shared drug-disease targets. Connections among them were shown by lines. Using Network Analyzer, core drug components were identified.

**Figure 4.**
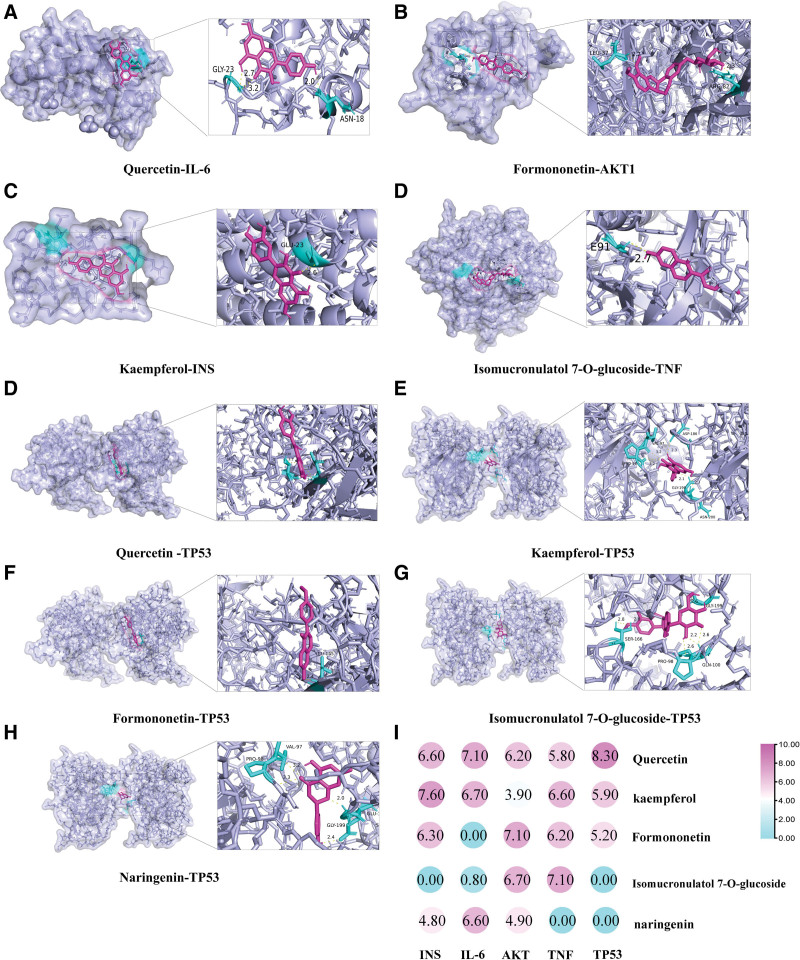
(A–H) Molecular docking of key active components of Beiqishen Jiangtang Granule with core targets (Total Score). (I) Schematic of molecular docking structures.

Based on the ranking of degree values of active ingredients and the criterion that most compounds exhibit multi-target interactions, 5 key active compounds and 5 core targets were selected for molecular docking (Fig. 3A). A total of 9 compound-target pairs with a Total Score > −7 kcal/mol were retained for visualization and analysis, including quercetin-IL-6, quercetin-TP53, kaempferol-INS, kaempferol-TP53, formononetin-AKT1, formononetin-TP53, isomucronulatol 7-*O*-glucoside-TNF-α, isomucronulatol 7-*O*-glucoside-TP53, and naringenin-TP53 (Fig. 4).

### 3.5. Effects of Beiqishen Jiangtang Granule on weight, water intake, and food intake in T2DM rats

**Figure 5.**
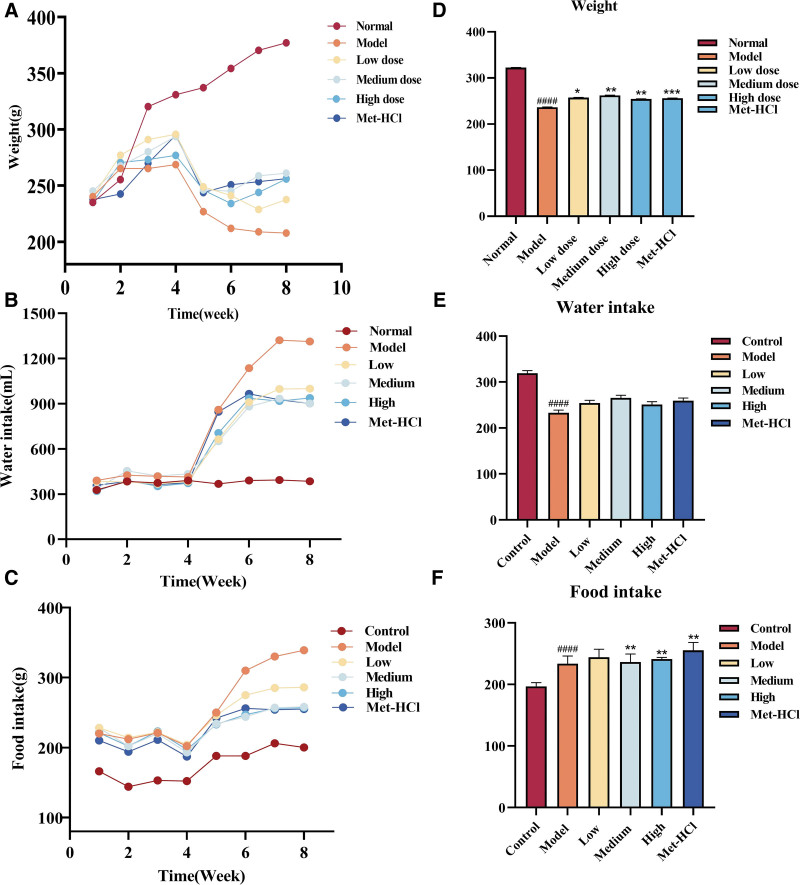
(A) Changes in body mass (g) of rats from 1 to 8 weeks (n = 8). (B) Changes in water intake (mL) of rats from 1 to 8 weeks (n = 8). (C) Changes in food intake (g) of rats for 1 to 8 weeks (n = 8). (D) Weight of each group of rats after administration. Water intake of rats in each group. (F) Food intake of each group of rats after administration. #### signifies *P* < .0001 vs the control cohort. * denotes *P* < .05 vs the model cohort; ** denotes *P* < .01 vs the model cohort; *** denotes *P* < .001 vs the model cohort. The same comparison criteria apply hereinafter.

In comparison to the normal cohort, rats in the model cohort presented a notable decline in weight (*P* < .0001), while their water and food intake were distinctly elevated (*P* < .0001). As opposed to the model cohort, rats in the low-, medium-, and high-dose cohorts of Beiqishen Jiangtang Granule and the metformin hydrochloride cohort displayed significant weight gain (*P* < .05). Nevertheless, the medium-dose and high-dose cohorts of Beiqishen Jiangtang Granule and the metformin hydrochloride cohort showed considerable decreases in food intake (*P* < .05). No notable discrepancies in water intake were found between the drug-treated cohorts and the model cohort (*P* > .05; Fig. 5).

### 3.6. Effects of Beiqishen Jiangtang Granule on fasting blood glucose and glucose tolerance in T2DM rats

**Figure 6.**
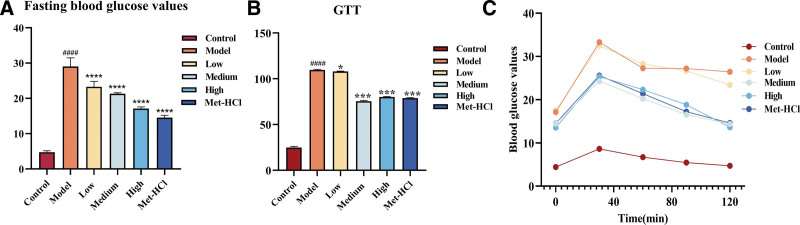
(A) Fasting blood glucose values of rats after administration. (B) Trend of blood glucose change within 2 hours of GTT. (C) Area under the GTT curve. #### signifies *P* < .0001 vs the control cohort. * denotes *P* < .05 vs the model cohort; *** denotes *P* < .001 vs the model cohort; **** denotes *P* < .0001 vs the model cohort. GTT = glucose tolerance test.

Relative to the normal control cohort, rats in the model cohort exhibited a significant elevation in fasting blood glucose concentrations (*P* < .0001). In contrast to the model cohort, rats in the low-, medium-, and high-dose cohorts of Beiqishen Jiangtang Granule and the metformin hydrochloride cohort demonstrated a marked reduction in fasting blood glucose concentrations (*P* < .0001; Fig. 6A).

The glucose tolerance test was employed to evaluate the organism’s capacity to regulate blood glucose. As illustrated in Figure 6B, C, relative to the normal cohort, rats in the model cohort showed a slower rate of blood glucose decline and a significantly expanded AUC (*P* < .0001), indicating impaired blood glucose regulatory ability. Conversely, compared with the model cohort, rats in the drug-treated cohorts exhibited more rapid restoration of blood glucose levels and a significantly decreased AUC (*P* < .05), suggesting enhanced blood glucose regulatory capacity.

### 3.7. Effects of Beiqishen Jiangtang Granule on liver and kidney indices

**Figure 7.**
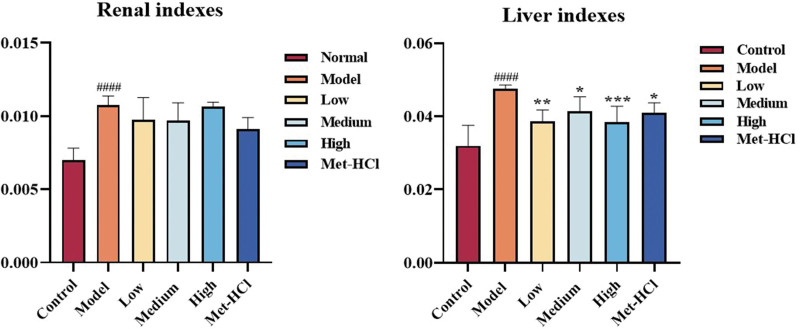
Renal and hepatic indices of rats across cohorts (*x* ± *s*, n = 8). #### signifies *P* < .0001 vs the control cohort. * denotes *P* < .05 vs the model cohort; ** denotes *P* < .01 vs the model cohort; *** denotes *P* < .001 vs the model cohort.

Relative to the normal cohort, the model cohort demonstrated marked elevations in both hepatic and renal indices (*P* < .0001), indicating statistically significant disparities. Relative to the model cohort, the low-, medium-, and high-dose cohorts of Beiqishen Jiangtang Granule and the metformin hydrochloride cohort exhibited notable reductions in hepatic index (*P* < .05). Additionally, the metformin hydrochloride cohort exhibited a notable decline in renal function index (*P* < .05). However, no notable disparities were observed in renal index across the low-, medium-, and high-dose cohorts of Beiqishen Jiangtang Granule (Fig. 7).

### 3.8. Effect of Beiqishen Jiangtang Granule on biochemical indexes of blood lipids in T2DM rats

**Figure 8.**
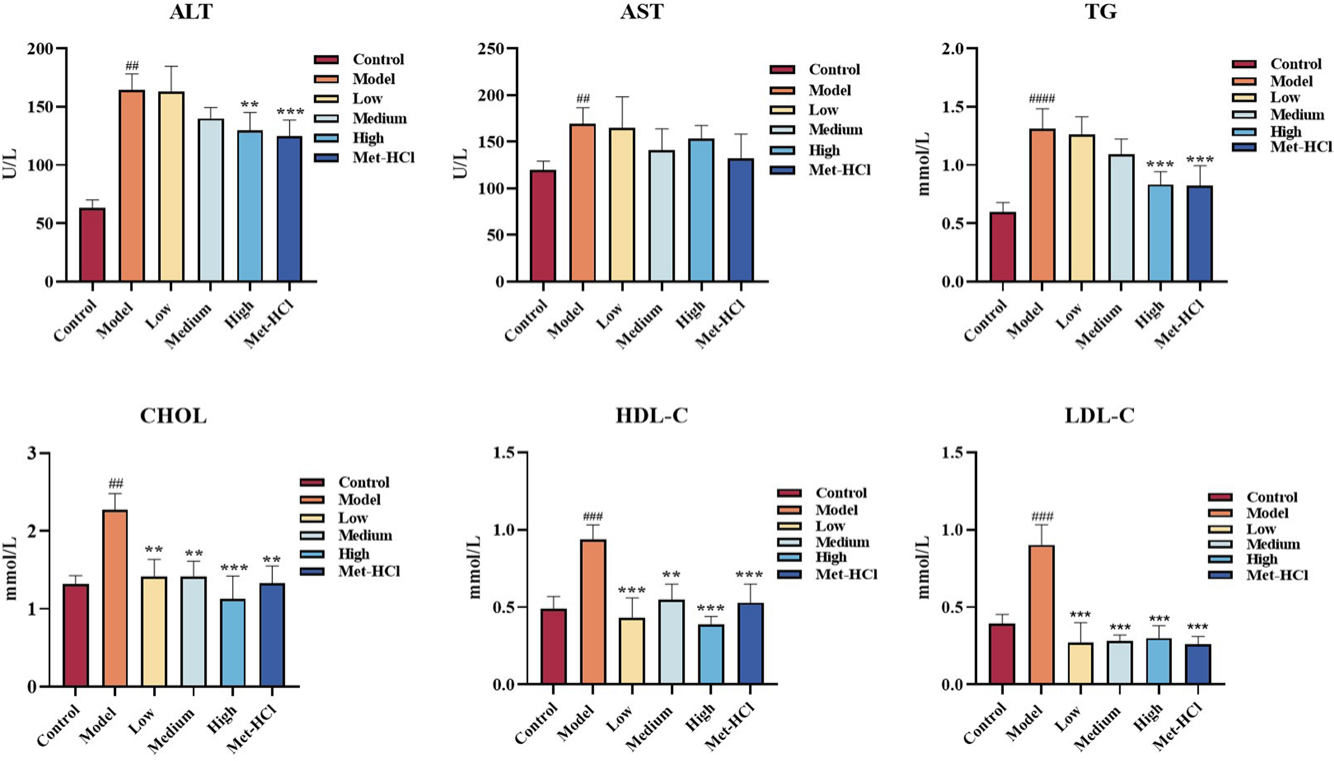
Biochemical indices of rats across cohorts (*x* ± *s*, n = 8). ## signifies *P* < .01 vs the control cohort; ### signifies *P* < .001 vs the control cohort; #### signifies *P* < .0001 vs the control cohort. ** denotes *P* < .01 vs the model cohort; *** denotes *P* < .001 vs the model cohort . ALT = alanine aminotransferase, AST = aspartate, CHOL = cholesterol, HDL-C = high-density lipoprotein cholesterol, LDL-C = low-density lipoprotein cholesterol, TG = triglycerides.

Relative to the normal cohort, serum levels of ALT, AST, TG, CHOL, LDL-C, and HDL-C in model group rats were markedly increased (*P* < .05). Relative to the model cohort, the high-dose Beiqishen Jiangtang Granule cohort and the metformin hydrochloride cohort showed notably decreased serum ALT and TG levels (*P* < .01). However, serum AST levels showed no notable disparity between drug-treated cohorts and the model cohort (*P* > .05). Additionally, serum CHOL, HDL-C, and LDL-C levels in drug-treated cohorts were markedly lower than those in the model cohort (*P* < .01; Fig. 8).

### 3.9. Effects of Beiqishen Jiangtang Granule on Ins, TNF-α, and IL-6 in T2DM rats

**Figure 9.**
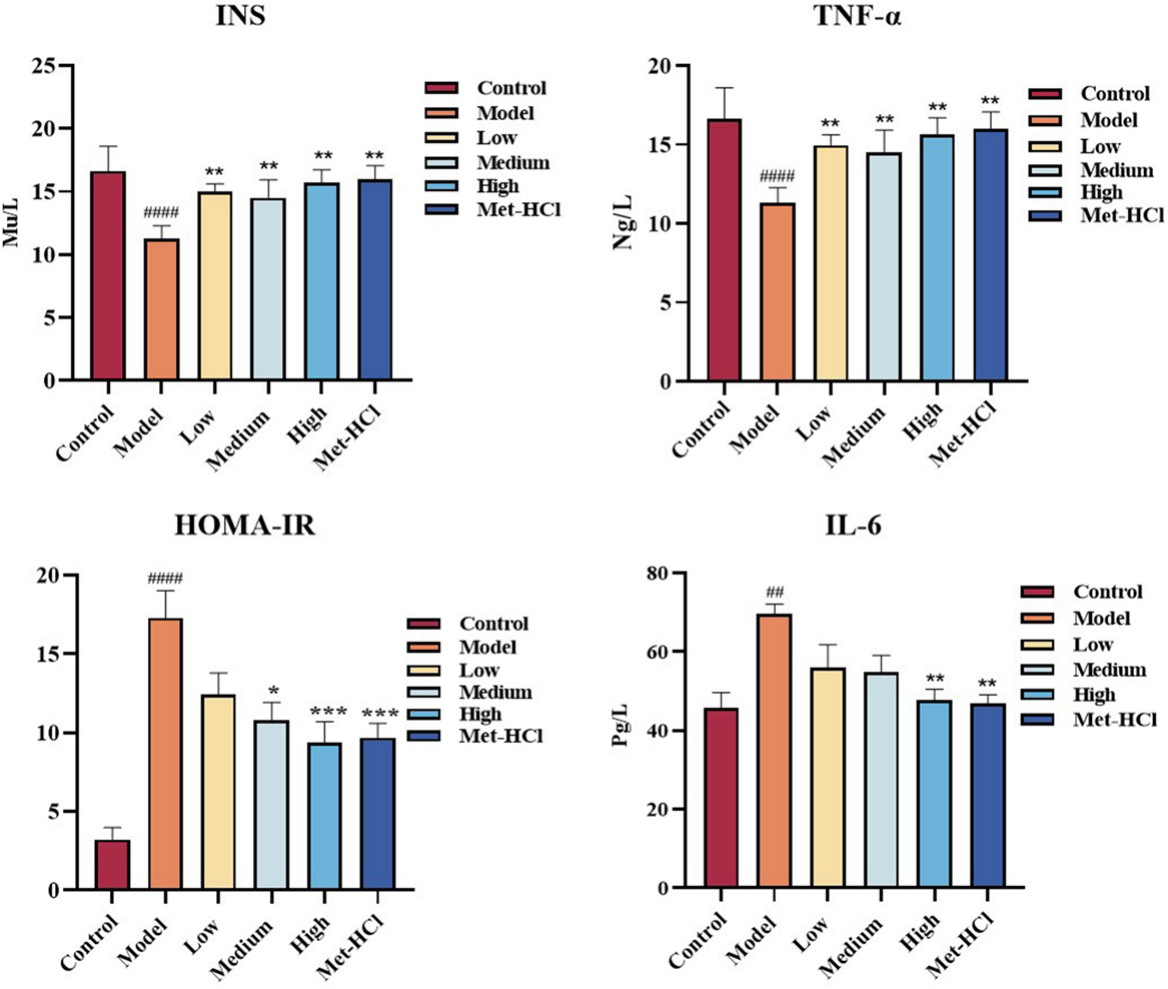
Contents of INS, TNF-α, IL-6 and HOMA-IR in serum of rats in each group (*x* ± *s*, n = 8). ## signifies *P* < .01 vs the control cohort; #### signifies *P* < .0001 vs the control cohort. * denotes *P* < .05 vs the model cohort; ** denotes *P* < .01 vs the model cohort; *** denotes *P* < .001 vs the model cohort. HOMA-IR = homeostasis model assessment of insulin resistance, INS = insulin, IL-6 = interleukin-6, TNF-α = tumor necrosis factor-alpha.

Relative to the normal cohort, the model cohort demonstrated a marked reduction in serum Ins levels (*P* < .0001). Postdrug administration, relative to the model cohort, the metformin hydrochloride cohort exhibited a notable elevation in serum Ins levels (*P* < .01). Additionally, the low-, medium-, and high-dose cohorts of Beiqishen Jiangtang Granule also showed marked elevations in Ins levels (*P* < .01). This suggests that both the positive drug and various concentrations of Beiqishen Jiangtang Granule can increase serum Ins levels to alleviate DM. The HOMA-IR calculated from Ins further supports the notion that both the positive drug and different concentrations of Beiqishen Jiangtang Granule have the potential to alleviate DM (Fig. 9A, B).

Relative to the control cohort, the model cohort demonstrated a marked elevation in TNF-α levels (*P* < .0001). Postdrug administration, relative to the model cohort, the metformin hydrochloride cohort exhibited a notable decline in serum TNF-α levels (*P* < .01). Additionally, the low-, medium-, and high-dose cohorts of Beiqishen Jiangtang Granule also showed marked reductions in TNF-α levels (*P* < .01). This suggests that both the positive drug and different concentrations of Beiqishen Jiangtang Granule can effectively reduce serum TNF-α levels to alleviate DM (Fig. 9C). Relative to the control cohort, the model cohort demonstrated a marked elevation in IL-6 levels (*P* < .01). Postdrug administration, relative to the model cohort, the metformin hydrochloride cohort exhibited a notable decline in serum IL-6 levels (*P* < .01). Moreover, the high-dose cohort of Beiqishen Jiangtang Granule also showed a marked reduction in IL-6 levels (*P* < .01). This indicates that both the positive drug and Beiqishen Jiangtang Granule can reduce serum IL-6 levels to alleviate DM, with the high-dose group of Beiqishen Jiangtang Granule showing superior effects compared to the low and medium-dose groups (Fig. 9D).

### 3.10. Effects of Beiqishen Jiangtang Granule on TP53 mRNA expression in T2DM rats

**Figure 10.**
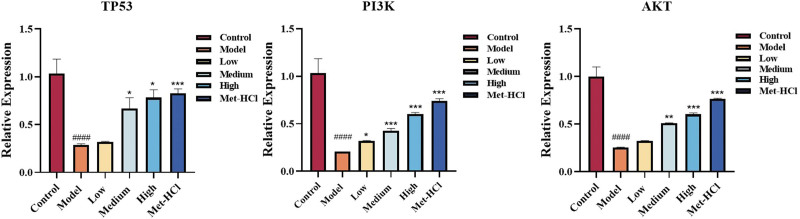
Relative expression of TP53mRNA, PI3KmRNA, and AkTmRNA in rat liver. #### signifies *P* < .0001 vs the control cohort. * denotes *P* < .05 vs the model cohort; ** denotes *P* < .01 vs the model cohort; *** denotes *P* < .001 vs the model cohort .

Relative to the model cohort, Akt protein expression was notably upregulated in the low-dose Beiqishen Jiangtang Granule cohort (*P* < .05). Conversely, TP53, Akt, and PI3K protein expression was also notably upregulated in the medium- and high-dose cohorts, along with the positive drug cohort (*P* < .01). TP53, PI3K, and Akt mRNA expression levels were notably elevated in the metformin hydrochloride cohort and the medium- and high-dose Beiqishen Jiangtang Granule cohorts (*P* < .05 or *P* < .01). The recovery degrees of the medium- and high-dose cohorts were comparable, exhibiting superior effects to the low-dose cohort. Among these cohorts, the metformin hydrochloride cohort exhibited expression levels most proximate to normal levels. These findings suggest that Beiqishen Jiangtang Granule modulates the T2DM-induced downregulation of TP53, Akt, and PI3K protein and mRNA expression (Fig. 10).

### 3.11. Western-blot indicators

**Figure 11.**
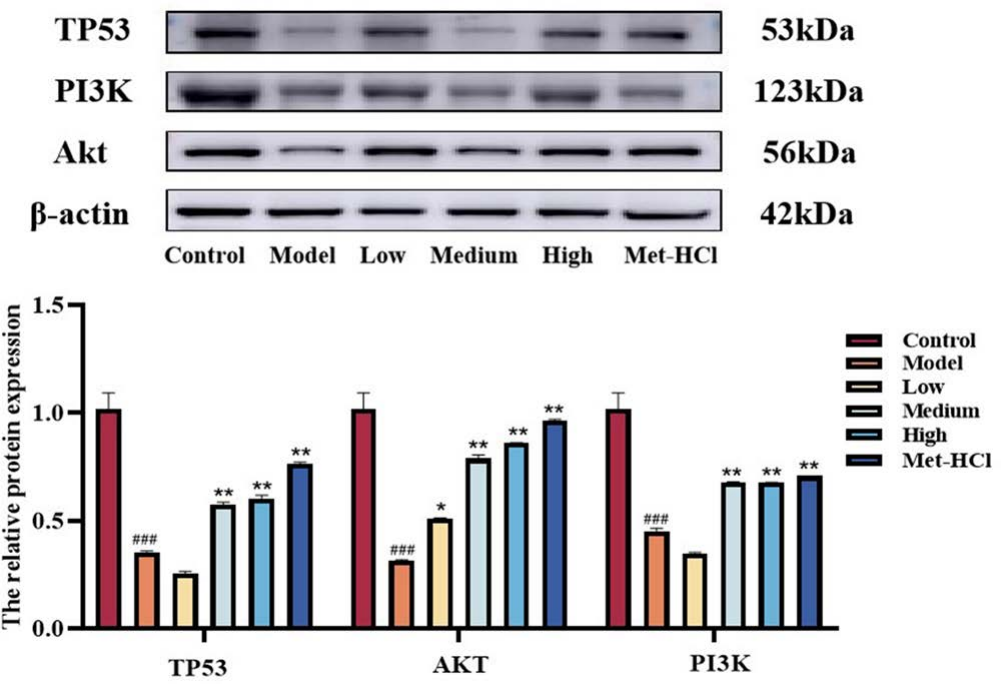
Relative expression of TP53, Akt, and PI3K proteins in rat hepatic tissue. ### signifies *P* < .001 vs the control cohort. * denotes *P* < .05 vs the model cohort; ** denotes *P* < .01 vs the model cohort .

Relative to the blank cohort, the expression levels of TP53, Akt, and PI3K proteins in the hepatic tissue of model group rats were notably decreased (*P* < .001). Relative to the model cohort, Akt protein expression was notably upregulated in the low-dose Beiqishen Jiangtang Granule cohort (*P* < .05). Meanwhile, TP53, Akt, and PI3K protein expression was also notably upregulated in the medium- and high-dose cohortsas in the positive drug cohort (*P* < .01). These findings suggest that Beiqishen Jiangtang Granule modulates the T2DM-induced downregulation of TP53, Akt, and PI3K protein expression (Fig. 11).

### 3.12. Findings of rat gut microbiota analysis

#### 3.12.1. Statistics of sequencing results

This study was divided into 6 groups: blank cohort, model cohort, positive drug cohort (metformin hydrochloride cohort), low-dose cohort of Beiqishen Jiangtang Granule, medium-dose cohort, and high-dose cohort, with 3 biological replicates per cohort, totaling 18 samples. The experiment generated a total of 23,92,890 raw paired-end reads (RawPE), averaging 1,32,938 reads per sample. After filtering, 20,18,142 valid non-chimeric reads (Nochime) were obtained, averaging 1,12,119 per sample, with a high-quality control efficiency of 84.42%. Among the valid data, over 97% of bases had a quality score > 20, over 93% had a quality score > 30, and the GC content exceeded 52%. The data for each group are as follows: The blank group yielded 4,13,953 RawPE reads, with 3,55,304 valid reads (average 1,18,435 per sample) and a quality control efficiency of 85.96%. The model group yielded 4,02,534 RawPE reads, with 3,43,705 valid reads (average 1,14,568 per sample) and a quality control efficiency of 85.41%. The positive drug group yielded 3,74,725 RawPE reads, with 3,26,647 valid reads (average 1,08,882 per sample) and a quality control efficiency of 87.46%. The low-dose group of Beiqishen Jiangtang Granule yielded 4,00,766 RawPE reads, with 3,49,733 valid reads (average 1,16,578 per sample) and a quality control efficiency of 87.27%. The medium-dose group yielded 4,15,796 RawPE reads, with 3,37,952 valid reads (average 1,12,651 per sample) and a quality control efficiency of 81.31%. The high-dose group yielded 3,85,116 RawPE reads, with 3,04,801 valid reads (average 1,01,600 per sample) and a quality control efficiency of 79.09% (Table S3, supplemental Digital Content, https://links.lww.com/MD/R301).

#### 3.12.2. Alpha diversity analysis, species accumulation boxplot analysis, and curve analysis of sample level clustering

**Figure 12.**
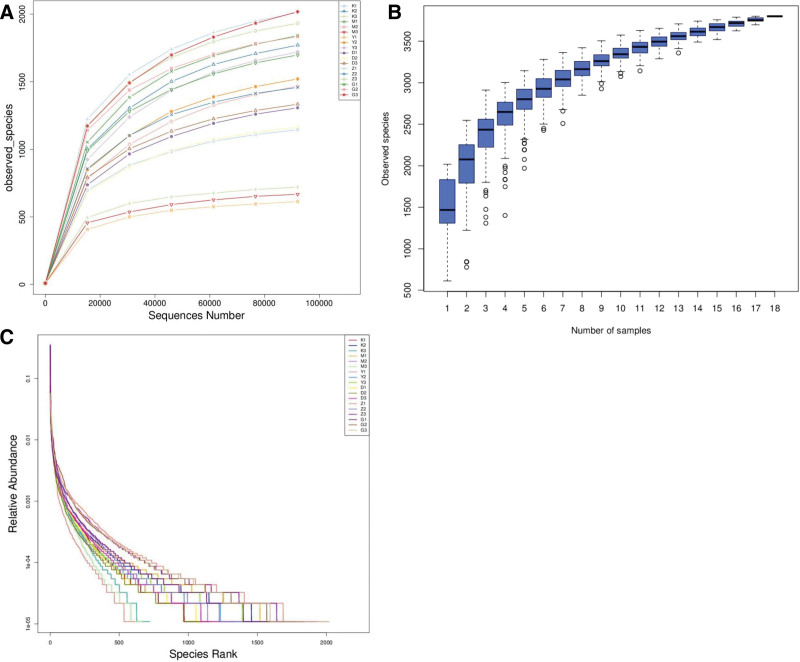
(A) Dilution curves. (B) Box plot depicting alpha diversity species accumulation. (C) Alpha diversity rank clustering curves.

The rarefaction curves indicate that species diversity initially increases rapidly with increasing sequencing depth. As the sequencing depth approaches 80,000 reads, the curves gradually plateau, indicating that species count ceases to rise substantially. This denotes that existing sequencing depth and sample quantity are adequate for the subsequent analyses. Additionally, the medium-dose group exhibited higher species richness, whereas the positive drug group exhibited lower species richness (Fig. 12A). In this study, 6 groups of samples (including the blank group) were analyzed. As illustrated in Figure 12B, as sample number increases, the boxplot gradually flattens, signifying that existing sample quantity suffices to cover most species and is credible for further research.

The rank-abundance curve serves to assess the evenness and richness of species in samples. The curve plots the rank of feature sequences (sorted by abundance) along the horizontal axis and relative abundance along the vertical axis. A more uniform species distribution results in a flatter curve, while a higher species richness results in a wider extent of the curve along the horizontal axis. As depicted in Figure 12, the curve for the positive drug group disappears first on the horizontal axis, while the curve of the medium-dose group disappears last and declines more slowly. The remaining 3 groups are positioned in the middle. The results indicate that the positive drug group has the lowest species abundance, while the medium-dose group has the highest abundance (Fig. 12C).

#### 3.12.3. Beta diversity analysis

**Figure 13.**
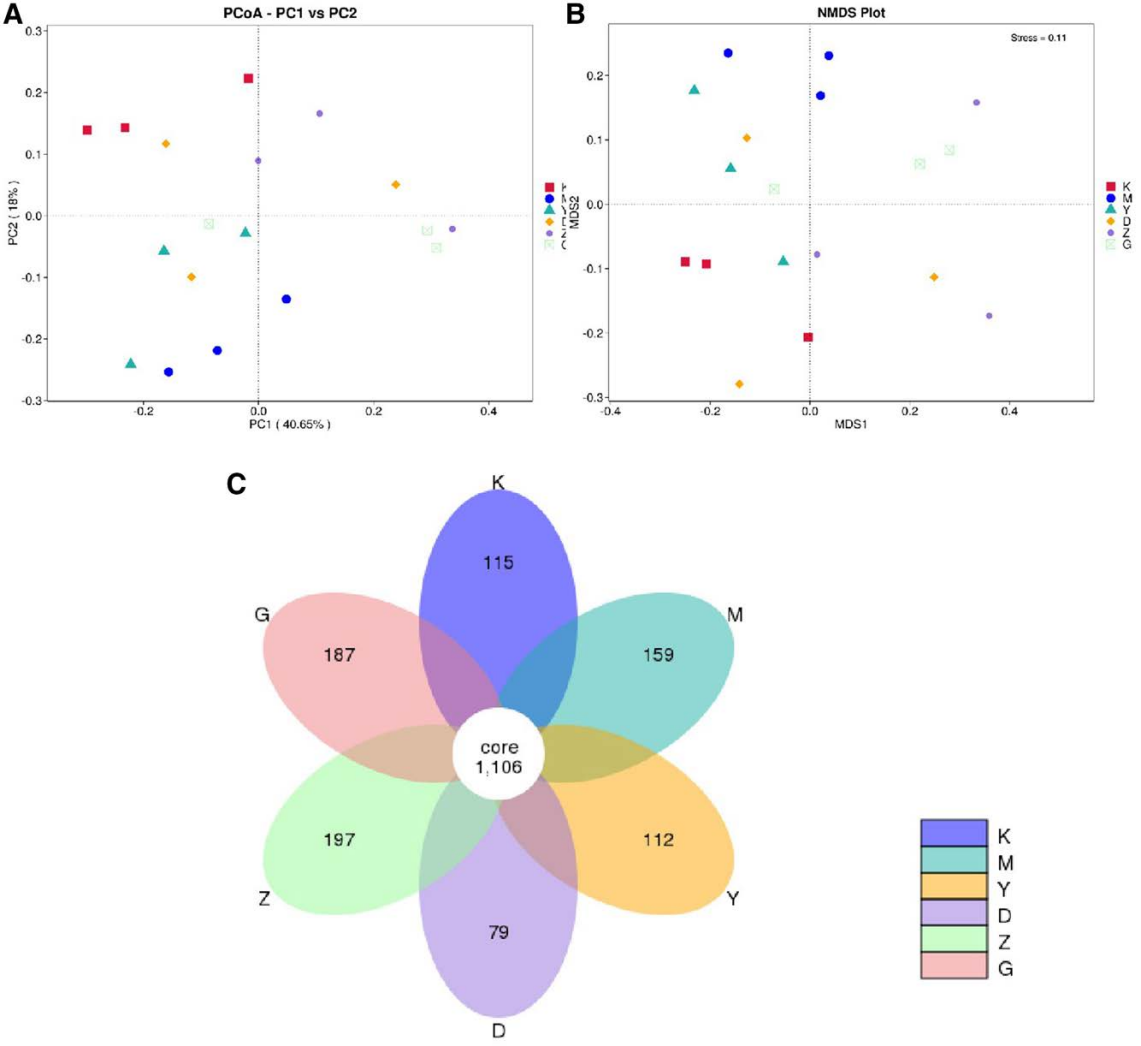
(A) Two-dimensional PCoA diagram. (B) NMDS plot. (C) Petal diagram species relative abundance and analysis. NMDS = nonmetric multidimensional scaling, PCoA = Principal Coordinates Analysis.

Principal Coordinates Analysis (PCoA) represents a technique that discerns the most pivotal components and architectures by performing a series of eigenvalue and eigenvector sorting on multidimensional data. In this study, Weighted UniFrac distance was used as the basis for PCoA analysis, and the results are shown in Figure 13A. It is not difficult to observe that samples from the same group are relatively clustered, while the gut microbiota of rats from different groups can be significantly separated. The positive drug cohort and the low-, medium-, and high-dose regimens of Beiqishen Jiangtang Granule are closer to the normal group. This result indicates that the gut microbiota structure of DM rats has changed, and the drug treatment has a notable influence on the diversity of the gut microbiota of DM rats, with a crtain regulatory effect.

Nonmetric multidimensional scaling (NMDS) is a nonmetric multidimensional scaling method, similar to PCoA analysis, and is also a sorting technique based on the distance matrix of samples (any type of distance). This figure illustrates the overall diversity changes of microbial communities across various groups, whereas no notable disparities exist among samples within identical groups, indicating strong representativeness (Fig. 13B). After normalizing and clustering the OTUs of all valid data from the 6 groups (blank, model, positive drug, alongside low-, medium-, and high-dose regimens of Beiqishen Jiangtang Granule), a petal diagram was used for analysis, as shown in Figure 13C The nonoverlapping parts in the figure represent the unique OTUs of each group, while the overlapping parts represent the shared OTUs between different groups. The figure shows that the 6 groups (blank, model, positive drug, alongside low-, medium-, and high-dose regimens of Beiqishen Jiangtang Granule) share a total of 1106 OTUs, with unique OTUs of 115, 159, 112, 79, 197, and 187, respectively.

#### 3.12.4. Gut microbiota composition and distribution analysis

Leveraging the annotation outcomes of species across diverse taxonomic ranks, we chose the top 10 species exhibiting the greatest relative prevalence in each sample cohort at every taxonomic rank and labeled the remaining species as others. Subsequently, we developed bar graphs illustrating relative prevalence for each sample cohort across diverse taxonomic ranks and utilized 1-way ANOVA to assess disparities in the relative prevalence of bacterial taxa at the phylum and genus ranks.

**Figure 14.**
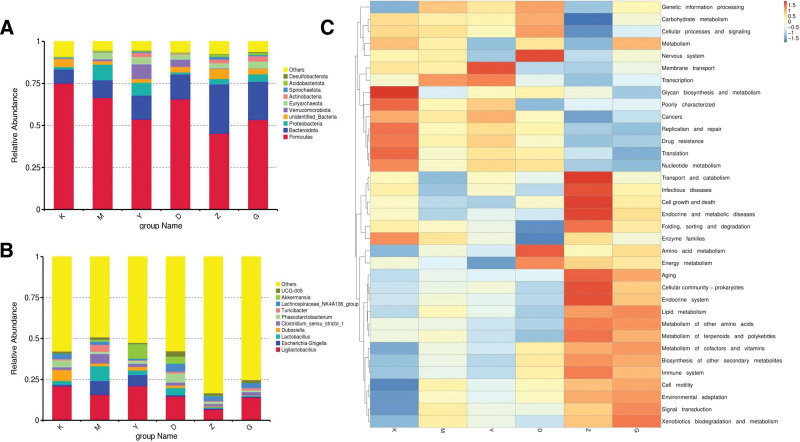
(A) Bar plots of relative abundance at the phylum level in rats across different treatment cohorts. (B) Column plots of genus-level relative abundance of rats in different treatment cohorts. (C) Tax4Fun functional prediction cluster heatmap.

As illustrated in Figure 14A at the phylum rank, the main species with relatively high abundance in the gut microbiota of rats across different groups include Firmicutes, Bacteroidota, Proteobacteria, unidentified_Bacteria, Verrucomicrobiota, Euryarchaeota, Actinobacteriota, Spirochaetota, Acidobacteriota, and Desulfobacterota. As shown in Figure 14B, at the genus rank, the predominant species with comparatively high prevalence in the gut microbiota of rats across the 6 groups include Ligilactobacillus, Escherichia-Shigella, Lactobacillus, Dubosiella, Clostridium_sensu_stricto_1, Phascolarctobacterium, Turicibacter, Lachnospiraceae_NK4A136_group, Akkermansia, and UCG-005.

The functional characteristics of the rat gut microbiota were forecasted via Tax4Fun, and a heatmap illustrating relative abundance was constructed, as depicted in Figure 14C. Within the blank cohort, the function of Glycan biosynthesis and metabolism was comparatively prominent. Within the model cohort, the function of Translation was comparatively elevated. Within the positive drug cohort, the function of Membrane transport was comparatively elevated. In the different dose groups of Beiqishen Jiangtang Granule, the functions of amino acid metabolism, cell growth and death, metabolism of cofactors and vitamins, nervous system, transport and breakdown, and xenobiotics biodegradation and metabolism were relatively high.

## 4. Discussion

The treatment of DM currently primarily relies on Western medicine, such as oral hypoglycemic agents and INS, which are often accompanied by side effects.^[4]^ In contrast, TCM treats DM through syndrome differentiation and the use of plant medicine, offering higher safety and a comprehensive therapeutic effect that addresses both symptoms and root causes, demonstrating good development potential.^[22]^ In Beiqishen Jiangtang Granule, *A membranaceus* serves as the principal herb, with functions including replenishing qi and uplifting yang, strengthening defensive qi and consolidating the exterior, nourishing blood and unblocking bi, and detoxifying and promoting tissue regeneration.^[23]^ *P ginseng*, which can nourish yin and generate body fluids as well as activate activate enhance blood circulation and dispel stasis, representing a frequently utilized herb within TCM for treating DM.^[24]^ *S chinensis* can replenish the liver and regulate the spleen, and mitigate the toxicity of hyperglycemia, being used for the treatment of diabetes insipidus.^[25]^ *G uralensis* is frequently used in both ancient and modern prescriptions for DM treatment and has shown significant therapeutic effects on DM.^[26]^. This study employed the method of network analysis to construct a molecular interaction network of Beiqishen Jiangtang Granule, predicting its main metabolites and involved signaling pathways for treating DM, thus taking an important step in exploring its therapeutic mechanisms in depth. The PPI network analysis of drug metabolites, targets, and signaling pathways revealed that the main metabolites of Beiqishen Jiangtang Granule for treating DM include quercetin, kaempferol, and formononetin. Quercetin is a widely distributed natural flavonol compound.

Owing to its anti-inflammatory, antioxidative, and immunomodulatory characteristics, many researchers have employed it in fundamental DM therapeutic investigations and have achieved certain results.^[27]^ Some studies have confirmed that quercetin can elicit hypoglycemic effects via modulation of the miR-92b-3p/EGR1 axis.^[28]^ Kaempferol has garnered extensive recognition for its antibacterial, anti-inflammatory, antioxidative, and antitumor activities.^[29]^ Studies have shown that kaempferol helps alleviate oxidative stress, inflammatory responses, and myocardial damage in diabetic rats.^[30]^ Formononetin is a polyphenolic nonsteroidal phytochemical with estrogenic activity, widely distributed in many plants, such as *A membranaceus* and Millettia.^[31]^ Formononetin has many biological properties, mainly manifested in its antitumor, antioxidant, anti-inflammatory, and anti-atherosclerosis capabilities. It has also been reported to have certain therapeutic effects on diabetes.^[32–34]^

Network pharmacology analysis identified 121 major metabolites (e.g., quercetin, kaempferol, formononetin) and 30 key targets (e.g., AKT1, TNF-α, IL-6, TP53, VEGFA, CASP3) of Beiqishen Jiangtang Granule (BQSGJT). GO/KEGG enrichment analyses revealed BQSGJT exerts hypoglycemic effects by regulating hormone levels, mediating cellular responses to lipids/organic substances, and modulating kinase binding/oxidoreductase activity in lipid rafts/transcriptional regulatory complexes, ultimately targeting lipid metabolism, PI3K-AKT, and FoxO signaling pathways. ELISA assays showed that compared with the blank group, T2DM model rats exhibited significant abnormalities in serum INS, TNF-α, IL-6, and HOMA-IR (INS resistance index). Notably, BQSGJT treatment elevated Ins levels, reduced TNF-α/IL-6 concentrations, and improved HOMA-IR (all statistically significant). Ins, a pancreatic β-cell-derived hormone, promotes glucose absorption/utilization/storage, while its deficiency/resistance induces hyperglycemia and T2DM.^[35,36]^ HOMA-IR, calculated via fasting blood glucose and Ins, reflects INS secretion capacity and predicts diabetic complication risk.^[37]^ TNF-α, an immune/cancer cell-derived cytokine, bidirectionally regulates glycemia: high levels enhance INS resistance, fatty acid release, and hepatic glycogen breakdown, thereby elevating blood glucose^[38]^

IL-6 represents a distinct cytokine secreted by diverse cell types, exerting a pivotal function in multiple aspects, encompassing inflammatory reactions, immune system modulation, as well as the control of metabolic balance.^[39]^ There is a specific connection between IL-6 and the reduction of blood glucose levels. High levels of IL-6 may induce Ins resistance, but adjusting the effects of IL-6 on fatty acid secretion may help reduce blood glucose levels. The close relationship between IL-6 and blood glucose control still needs further exploration. This study suggests that Beiqishen Jiangtang Granule may lower blood glucose levels by regulating Ins, TNF-α, and IL-6. Additionally, molecular docking and experimental validation have demonstrated that these compounds have strong binding affinities with core targets and hold potential therapeutic effects in improving Ins resistance and diabetic complications. These results offer theoretical underpinnings for the multi-target mode of action of Beiqishen Jiangtang Granule and lay the foundation for further clinical research.

This study demonstrates that Beiqishen Jiangtang Granule administration significantly upregulates the hepatic protein and mRNA expression of TP53, PI3K, and Akt in T2DM rats, which tightly links to the core PI3K-Akt pathway implicated in diabetic pathogenesis. As a key downstream molecule of the PI3K-Akt pathway, TP53 regulates pancreatic islet cell function and glucose metabolism through multiple mechanisms^[40]^: it modulates INS secretion, alleviates islet cell apoptosis, and regulates glucose transporter activity, with its aberrant activation contributing to reduced Ins secretion, impaired cellular glucose uptake, and subsequent Ins resistance and hyperglycemia.^[41]^ Consistent with our computational predictions, these experimental findings confirm that Beiqishen Jiangtang Granule exerts hypoglycemic effects by targeting the PI3K-Akt pathway (via regulating TP53, PI3K, and Akt expression) and modulating downstream effector factors including Ins, TNF-α, and IL-6, fully validating the consistency between in silico predictions and in vivo experimental data. It can be seen from the literature database that Chinese medicine has paid extensive attention to the use of *P ginseng* to improve intestinal flora in the treatment of diabetes, and has conducted research grounded in molecular pathway frameworks.^[42]^ Findings from this investigation illustrate that the sample size was sufficiently large and the data were robust, ensuring that the sequencing outcomes accurately reflected the true conditions of the samples.^[43]^ Alpha diversity analysis indicated that, when juxtaposed against the blank group, the evenness and richness of gut microbiota in other cohorts exhibited no marked alterations. However, beta diversity analysis revealed that therapeutic intervention with Beiqishen Jiangtang Granule exerted a pronounced regulatory effect on the gut microbiota diversity of rats with T2DM. Additionally, KEGG pathway enrichment analysis identified the PI3K-Akt signaling pathway as a key molecular target through which Beiqishen Jiangtang Granule ameliorates T2DM. ^[44]^

The study suggests that Beiqishen Jiangtang Granule may orchestrate the compositional dynamics of gut microbiota across multiple dimensions, exerting a dual-regulatory effect: it fosters the proliferation of probiotic taxa (e.g., Ligilactobacillus, Lachnospiraceae_NK4A136_group) while concurrently dampening the prevalence of pathogenic microbial components, including Lactobacillus, Clostridium sensu stricto 1, and Turicibacter. By rebalancing the intestinal microecology through this pathway, Beiqishen Jiangtang Granule may confer therapeutic efficacy against T2DM.

## 5. Conclusion

Based on network pharmacology predictions, this study preliminarily elucidated Beiqishen Jiangtang Granule potential antidiabetic mechanisms. Notably, these in silico findings have limitations – relying on existing databases (failing to fully reflect in vivo multicomponent interactions) and ignoring individual drug response differences – thus requiring in vivo and clinical validation. The predictions indicate Beiqishen Jiangtang Granule exerts hypoglycemic effects via a multi-target, multi-pathway paradigm: regulating serum INS, TNF-α, IL-6; modulating TP53, Akt, PI3K (protein/mRNA levels); and balancing intestinal flora by increasing beneficial bacteria and reducing harmful ones. Future studies will verify these targets/pathways in T2DM animal models and conduct clinical trials to confirm Beiqishen Jiangtang Granule efficacy and safety for reliable clinical application.

## Author contributions

**Conceptualization:** Meitong Pan, Panpan Wang, Wei Ma.

**Data curation:** Meitong Pan, Panpan Wang, Zhen Wang, Zhanping Zhang.

**Formal analysis:** Xinxin Wang, Zhanping Zhang.

**Funding acquisition:** Keke Yang.

**Methodology:** Xinxin Wang, Weili Liu.

**Investigation:** Zhen Wang, Weili Liu.

**Resources:** Xiubo Liu.

**Software:** Keke Yang.

**Supervision:** Keke Yang.

## Supplementary Material


