# Supplementary material for: Insights into the potential mechanism of Beiqishen Jiangtang Granule in the treatment of type 2 diabetes nephropathy: A study based on network pharmacology, molecular docking, and biological validation
Source: Medicine (Baltimore). 2026 Feb 13;105(7):e47533. doi: 10.1097/MD.0000000000047533 (PMC12908841; doi:10.1097/MD.0000000000047533)
Supplement: Supplementary file 1 [file medi-105-e47533-s001.docx]

Supplementary Tables

**Supplement table 1 Primer information**

| **Name** | **Seq** |
| --- | --- |
| actin-F | TCTTCCAGCCTTCCTTCCTG |
| actin-R | CACACAGAGTACTTGCGCTC |
| TP53-F | CAGCCCTCTTTACCCTACCC |
| TP53-R | GGGCTCCATCCTGACTGTAA |
| PI3K-F | AAACTCCTCCAGCCTTCCTC |
| PI3K-R | TCCGGCTCTGACATGAACTT |
| AKT-F | CTGCCCTTCTACAACCAGGA |
| AKT-R | GTGCTGCATGATCTCCTTGG |

**Supplement table 2 The main active ingredients of Beiqishen**

| **Marker** | **Mol ID** | **Component** | **OB(％)** | **DL** | **Source** |
| --- | --- | --- | --- | --- | --- |
| HQ1 | MOL000033 | (3S,8S,9S,10R,13R,14S,17R) -10,13-dimethyl-17-[(2R,5S) -5-propan-2-yloctan-2-yl]- 2,3,4,7,8,9,11,12,14,15,16, 17-dodecahydro-1H- cyclopenta[a]phenanthren-3 -ol | 36.23 | 0.78 | *Astragalus membranaceus* |
| HQ2 | MOL000296 | hederagenin | 36.91 | 0.75 | *Astragalus membranaceus* |
| HQ3 | MOL000371 | 3,9-di-O-methylnissolin | 53.74 | 0.48 | *Astragalus membranaceus* |
| HQ4 | MOL000378 | 7-O-methylisomucronulatol | 74.69 | 0.3 | *Astragalus membranaceus* |
| HQ5 | MOL000379 | 9,10-dimethoxypterocarpan -3-O-β-D-glucoside | 36.74 | 0.92 | *Astragalus membranaceus* |
| HQ6 | MOL000380 | (6aR,11aR)-9,10-dimethoxy -6a,11a-dihydro-6H- benzofurano[3,2-c]chromen- 3-ol | 64.26 | 0.42 | *Astragalus membranaceus* |
| HQ7 | MOL000387 | Bifendate | 31.1 | 0.67 | *Astragalus membranaceus* |
| HQ8 | MOL000433 | FA | 68.96 | 0.71 | *Astragalus membranaceus* |
| HQ9 | MOL000439 | isomucronulatol-7,2'-di -O-glucosiole | 49.28 | 0.62 | *Astragalus membranaceus* |
| HQ10 | MOL000442 | 1,7-Dihydroxy-3,9- dimethoxy pterocarpene | 39.05 | 0.48 | *Astragalus membranaceus* |
| RS1 | MOL000358 | beta-sitosterol | 36.91 | 0.75 | *Panax ginseng* |
| RS2 | MOL000449 | Stigmasterol | 43.83 | 0.76 | *Panax ginseng* |
| RS3 | MOL000787 | Fumarine | 59.26 | 0.83 | *Panax ginseng* |
| RS4 | MOL002879 | Diop | 43.59 | 0.39 | *Panax ginseng* |
| RS5 | MOL003648 | Inermin | 65.83 | 0.54 | *Panax ginseng* |
| RS6 | MOL005308 | Aposiopolamine | 66.65 | 0.22 | *Panax ginseng* |
| RS7 | MOL005318 | Dianthramine | 40.45 | 0.2 | *Panax ginseng* |
| RS8 | MOL005320 | arachidonate | 45.57 | 0.2 | *Panax ginseng* |
| RS9 | MOL005321 | Frutinone A | 65.9 | 0.34 | *Panax ginseng* |
| RS10 | MOL005344 | ginsenoside rh2 | 36.32 | 0.56 | *Panax ginseng* |
| RS11 | MOL005348 | Ginsenoside-Rh4_qt | 31.11 | 0.78 | *Panax ginseng* |
| RS12 | MOL005356 | Girinimbin | 61.22 | 0.31 | *Panax ginseng* |
| RS13 | MOL005376 | Panaxadiol | 33.09 | 0.79 | *Panax ginseng* |
| RS14 | MOL005384 | suchilactone | 57.52 | 0.56 | *Panax ginseng* |
| RS15 | MOL005399 | alexandrin_qt | 36.91 | 0.75 | *Panax ginseng* |
| WWZ1 | MOL004624 | Longikaurin A | 47.72 | 0.53 | *Schisandra chinensis* |
| WWZ2 | MOL008956 | Angeloylgomisin O | 31.97 | 0.85 | *Schisandra chinensis* |
| WWZ3 | MOL008957 | Schizandrer B | 30.71 | 0.83 | *Schisandra chinensis* |
| WWZ4 | MOL008968 | Gomisin-A | 30.69 | 0.78 | *Schisandra chinensis* |
| WWZ5 | MOL008974 | Gomisin G | 32.68 | 0.83 | *Schisandra chinensis* |
| WWZ6 | MOL008978 | Gomisin R | 34.84 | 0.86 | *Schisandra chinensis* |
| WWZ7 | MOL008992 | Schisandra chinensissu C | 46.27 | 0.84 | *Schisandra chinensis* |
| GC1 | MOL000359 | sitosterol | 36.91 | 0.75 | *Glycyrrhiza uralensis* |
| GC2 | MOL000497 | licochalcone a | 40.79 | 0.29 | *Glycyrrhiza uralensis* |
| GC3 | MOL000500 | Vestitol | 74.66 | 0.21 | *Glycyrrhiza uralensis* |
| GC4 | MOL001484 | Inermine | 75.18 | 0.54 | *Glycyrrhiza uralensis* |
| GC5 | MOL001792 | DFV | 32.76 | 0.18 | *Glycyrrhiza uralensis* |
| GC6 | MOL002311 | Glycyrol | 90.78 | 0.67 | *Glycyrrhiza uralensis* |
| GC7 | MOL002565 | Medicarpin | 49.22 | 0.34 | *Glycyrrhiza uralensis* |
| GC8 | MOL003656 | Lupiwighteone | 51.64 | 0.37 | *Glycyrrhiza uralensis* |
| GC9 | MOL003896 | 7-Methoxy-2-methyl  isoflavone | 42.56 | 0.2 | *Glycyrrhiza uralensis* |
| GC10 | MOL004328 | naringenin | 59.29 | 0.21 | *Glycyrrhiza uralensis* |
| GC11 | MOL004805 | (2S)-2-[4-hydroxy-3- (3-methylbut-2-enyl)phenyl] -8,8-dimethyl-2,3- dihydropyrano[2,3-f]chromen -4-one | 31.79 | 0.72 | *Glycyrrhiza uralensis* |
| GC12 | MOL004806 | euchrenone | 30.29 | 0.57 | *Glycyrrhiza uralensis* |
| GC13 | MOL004808 | glyasperin B | 65.22 | 0.44 | *Glycyrrhiza uralensis* |
| GC14 | MOL004810 | glyasperin F | 75.84 | 0.54 | *Glycyrrhiza uralensis* |
| GC15 | MOL004811 | Glyasperin C | 45.56 | 0.4 | *Glycyrrhiza uralensis* |
| GC16 | MOL004814 | Isotrifoliol | 31.94 | 0.42 | *Glycyrrhiza uralensis* |
| GC17 | MOL004815 | (E)-1-(2,4-dihydroxyphenyl) -3-(2,2-dimethylchromen-6 -yl)prop-2-en-1-one | 39.62 | 0.35 | *Glycyrrhiza uralensis* |
| GC18 | MOL004820 | kanzonols W | 50.48 | 0.52 | *Glycyrrhiza uralensis* |
| GC19 | MOL004824 | (2S)-6-(2,4-dihydroxyphenyl) -2-(2-hydroxypropan-2-yl)-4- methoxy-2,3-dihydrofuro [3,2-g]chromen-7-one | 60.25 | 0.63 | *Glycyrrhiza uralensis* |
| GC20 | MOL004827 | Semilicoisoflavone B | 48.78 | 0.55 | *Glycyrrhiza uralensis* |
| GC21 | MOL004828 | Glepidotin A | 44.72 | 0.35 | *Glycyrrhiza uralensis* |
| GC22 | MOL004829 | Glepidotin B | 64.46 | 0.34 | *Glycyrrhiza uralensis* |
| GC23 | MOL004833 | Phaseolinisoflavan | 32.01 | 0.45 | *Glycyrrhiza uralensis* |
| GC24 | MOL004835 | Glypallichalcone | 61.6 | 0.19 | *Glycyrrhiza uralensis* |
| GC25 | MOL004838 | 8-(6-hydroxy-2-benzofuranyl) -2,2-dimethyl-5-chromenol | 58.44 | 0.38 | *Glycyrrhiza uralensis* |
| GC26 | MOL004841 | Licochalcone B | 76.76 | 0.19 | *Glycyrrhiza uralensis* |
| GC27 | MOL004848 | licochalcone G | 49.25 | 0.32 | *Glycyrrhiza uralensis* |
| GC28 | MOL004849 | 3-(2,4-dihydroxyphenyl)-8- (1,1-dimethylprop-2-enyl)-7 -hydroxy-5-methoxy-coumarin | 59.62 | 0.43 | *Glycyrrhiza uralensis* |
| GC29 | MOL004855 | Licoricone | 63.58 | 0.47 | *Glycyrrhiza uralensis* |
| GC30 | MOL004856 | Glycyrrhiza uralensisnin A | 51.08 | 0.4 | *Glycyrrhiza uralensis* |
| GC31 | MOL004857 | Glycyrrhiza uralensisnin B | 48.79 | 0.45 | *Glycyrrhiza uralensis* |
| GC32 | MOL004863 | 3-(3,4-dihydroxyphenyl)-5,7 -dihydroxy-8-(3-methylbut- 2-enyl)chromone | 66.37 | 0.41 | *Glycyrrhiza uralensis* |
| GC33 | MOL004864 | 5,7-dihydroxy-3- (4-methoxyphenyl)-8-(3- methylbut-2-enyl)chromone | 30.49 | 0.41 | *Glycyrrhiza uralensis* |
| GC34 | MOL004866 | 2-(3,4-dihydroxyphenyl)-5,7 -dihydroxy-6-(3-methylbut-2 -enyl)chromone | 44.15 | 0.41 | *Glycyrrhiza uralensis* |
| GC35 | MOL004879 | Glycyrin | 52.61 | 0.47 | *Glycyrrhiza uralensis* |
| GC36 | MOL004882 | Licocoumarone | 33.21 | 0.36 | *Glycyrrhiza uralensis* |
| GC37 | MOL004883 | Licoisoflavone | 41.61 | 0.42 | *Glycyrrhiza uralensis* |
| GC38 | MOL004884 | Licoisoflavone B | 38.93 | 0.55 | *Glycyrrhiza uralensis* |
| GC39 | MOL004885 | licoisoflavanone | 52.47 | 0.54 | *Glycyrrhiza uralensis* |
| GC40 | MOL004891 | shinpterocarpin | 80.3 | 0.73 | *Glycyrrhiza uralensis* |
| GC41 | MOL004898 | (E)-3-[3,4-dihydroxy-5- (3-methylbut-2-enyl)phenyl] -1-(2,4-dihydroxyphenyl) prop-2-en-1-one | 46.27 | 0.31 | *Glycyrrhiza uralensis* |
| GC42 | MOL004903 | liquiritin | 65.69 | 0.74 | *Glycyrrhiza uralensis* |
| GC43 | MOL004904 | licopyranocoumarin | 80.36 | 0.65 | *Glycyrrhiza uralensis* |
| GC44 | MOL004907 | Glyzaglabrin | 61.07 | 0.35 | *Glycyrrhiza uralensis* |
| GC45 | MOL004908 | Glabridin | 53.25 | 0.47 | *Glycyrrhiza uralensis* |
| GC46 | MOL004910 | Glabranin | 52.9 | 0.31 | *Glycyrrhiza uralensis* |
| GC47 | MOL004911 | Glabrene | 46.27 | 0.44 | *Glycyrrhiza uralensis* |
| GC48 | MOL004912 | Glabrone | 52.51 | 0.5 | *Glycyrrhiza uralensis* |
| GC49 | MOL004913 | 1,3-dihydroxy-9-methoxy-6 -benzofurano[3,2-c] chromenone | 48.14 | 0.43 | *Glycyrrhiza uralensis* |
| GC50 | MOL004914 | 1,3-dihydroxy-8,9-dimethoxy -6-benzofurano[3,2-c] chromenone | 62.9 | 0.53 | *Glycyrrhiza uralensis* |
| GC51 | MOL004915 | Eurycarpin A | 43.28 | 0.37 | *Glycyrrhiza uralensis* |
| GC52 | MOL004924 | (-)-Medicocarpin | 40.99 | 0.95 | *Glycyrrhiza uralensis* |
| GC53 | MOL004935 | Sigmoidin-B | 34.88 | 0.41 | *Glycyrrhiza uralensis* |
| GC54 | MOL004941 | (2R)-7-hydroxy-2- (4-hydroxyphenyl)chroman-4 -one | 71.12 | 0.18 | *Glycyrrhiza uralensis* |
| GC55 | MOL004945 | (2S)-7-hydroxy-2- (4-hydroxyphenyl)-8-(3- methylbut-2-enyl)chroman-4 -one | 36.57 | 0.32 | *Glycyrrhiza uralensis* |
| GC56 | MOL004948 | Isoglycyrol | 44.7 | 0.84 | *Glycyrrhiza uralensis* |
| GC57 | MOL004949 | Isolicoflavonol | 45.17 | 0.42 | *Glycyrrhiza uralensis* |
| GC58 | MOL004957 | HMO | 38.37 | 0.21 | *Glycyrrhiza uralensis* |
| GC59 | MOL004959 | 1-Methoxyphaseollidin | 69.98 | 0.64 | *Glycyrrhiza uralensis* |
| GC60 | MOL004961 | Quercetin der. | 46.45 | 0.33 | *Glycyrrhiza uralensis* |
| GC61 | MOL004966 | 3'-Hydroxy-4'-O- Methylglabridin | 43.71 | 0.57 | *Glycyrrhiza uralensis* |
| GC62 | MOL004974 | 3'-Methoxyglabridin | 46.16 | 0.57 | *Glycyrrhiza uralensis* |
| GC63 | MOL004978 | 2-[(3R)-8,8-dimethyl-3,4 -dihydro-2H-pyrano[6,5-f] chromen-3-yl]-5 -methoxyphenol | 36.21 | 0.52 | *Glycyrrhiza uralensis* |
| GC64 | MOL004980 | Inflacoumarin A | 39.71 | 0.33 | *Glycyrrhiza uralensis* |
| GC65 | MOL004985 | icos-5-enoic acid | 30.7 | 0.2 | *Glycyrrhiza uralensis* |
| GC66 | MOL004988 | Kanzonol F | 32.47 | 0.89 | *Glycyrrhiza uralensis* |
| GC67 | MOL004989 | 6-prenylated eriodictyol | 39.22 | 0.41 | *Glycyrrhiza uralensis* |
| GC68 | MOL004990 | 7,2',4'-trihydroxy－5 -methoxy-3－arylcoumarin | 83.71 | 0.27 | *Glycyrrhiza uralensis* |
| GC69 | MOL004991 | 7-Acetoxy-2-methylisoflavone | 38.92 | 0.26 | *Glycyrrhiza uralensis* |
| GC70 | MOL004993 | 8-prenylated eriodictyol | 53.79 | 0.4 | *Glycyrrhiza uralensis* |
| GC71 | MOL004996 | gadelaidic acid | 30.7 | 0.2 | *Glycyrrhiza uralensis* |
| GC72 | MOL005000 | Glycyrrhiza uralensisnin G | 60.44 | 0.39 | *Glycyrrhiza uralensis* |
| GC73 | MOL005001 | Glycyrrhiza uralensisnin H | 50.1 | 0.78 | *Glycyrrhiza uralensis* |
| GC74 | MOL005003 | Licoagrocarpin | 58.81 | 0.58 | *Glycyrrhiza uralensis* |
| GC75 | MOL005007 | Glyasperins M | 72.67 | 0.59 | *Glycyrrhiza uralensis* |
| GC76 | MOL005008 | Glycyrrhiza flavonol A | 41.28 | 0.6 | *Glycyrrhiza uralensis* |
| GC77 | MOL005012 | Licoagroisoflavone | 57.28 | 0.49 | *Glycyrrhiza uralensis* |
| GC78 | MOL005016 | Odoratin | 49.95 | 0.3 | *Glycyrrhiza uralensis* |
| GC79 | MOL005017 | Phaseol | 78.77 | 0.58 | *Glycyrrhiza uralensis* |
| GC80 | MOL005018 | Xambioona | 54.85 | 0.87 | *Glycyrrhiza uralensis* |
| GC81 | MOL005020 | dehydroglyasperins C | 53.82 | 0.37 | *Glycyrrhiza uralensis* |
| A1 | MOL000422 | kaempferol | 41.88 | 0.24 | *Panax ginseng、Astragalus membranaceus、 Glycyrrhiza uralensis* |
| B1 | MOL000098 | quercetin | 46.43 | 0.28 | *Astragalus membranaceus、Glycyrrhiza uralensis* |
| B2 | MOL000211 | Mairin | 55.38 | 0.78 | *Astragalus membranaceus、Glycyrrhiza uralensis* |
| B3 | MOL000239 | Jaranol | 50.83 | 0.29 | *Astragalus membranaceus、Glycyrrhiza uralensis* |
| B4 | MOL000354 | isorhamnetin | 49.6 | 0.31 | *Astragalus membranaceus、Glycyrrhiza uralensis* |
| B5 | MOL000392 | formononetin | 69.67 | 0.21 | *Astragalus membranaceus、Glycyrrhiza uralensis* |
| B6 | MOL000417 | Calycosin | 47.75 | 0.24 | *Astragalus membranaceus、Glycyrrhiza uralensis* |
| C1 | MOL005317 | Deoxyharringtonine | 39.27 | 0.81 | *Schisandra chinensis、Panax ginseng* |

**Supplement table 3 raw statistics of sequencing results**

| **Sample** | **RawPE** | **Nochime** | **Base(nt)** | **GC** | **Q20** | **Q30** | **Effective** |
| --- | --- | --- | --- | --- | --- | --- | --- |
| K1 | 132733 | 116648 | 48793648 | 52.71% | 97.86% | 93.39% | 87.88% |
| K2 | 145636 | 118274 | 49557311 | 53.17% | 98.04% | 93.78% | 81.21% |
| K3 | 135584 | 120382 | 50148026 | 52.98% | 98.17% | 94.11% | 88.79% |
| M1 | 133629 | 105353 | 43912161 | 53.45% | 98.15% | 94.14% | 78.84% |
| M2 | 138332 | 119950 | 50416609 | 52.64% | 97.82% | 93.32% | 86.71% |
| M3 | 130573 | 118402 | 49545268 | 52.91% | 98.07% | 93.90% | 90.68% |
| Y1 | 104222 | 94774 | 39788009 | 52.98% | 98.06% | 93.82% | 90.93% |
| Y2 | 133015 | 114049 | 47453655 | 53.52% | 98.23% | 94.25% | 85.74% |
| Y3 | 137488 | 117824 | 49422439 | 53.04% | 97.96% | 93.58% | 85.70% |
| D1 | 135617 | 118012 | 48730567 | 53.37% | 98.22% | 94.24% | 87.02% |
| D2 | 132282 | 116339 | 48940091 | 52.38% | 97.79% | 93.21% | 87.95% |
| D3 | 132867 | 115382 | 47850677 | 55.03% | 98.20% | 94.22% | 86.84% |
| Z1 | 136546 | 110980 | 45994885 | 53.48% | 98.14% | 94.07% | 81.28% |
| Z2 | 136028 | 113548 | 47619775 | 55.69% | 97.83% | 93.32% | 83.47% |
| Z3 | 143222 | 113424 | 46801069 | 53.23% | 98.23% | 94.27% | 79.19% |
| G1 | 133396 | 109406 | 45797571 | 52.77% | 97.76% | 93.13% | 82.02% |
| G2 | 127092 | 98767 | 40950600 | 53.53% | 98.08% | 93.94% | 77.71% |
| G3 | 124628 | 96628 | 39862979 | 53.89% | 98.18% | 94.19% | 77.53% |

**Base refers to the number of bases in the final Nochime data; GC (%) indicates the percentage of GC bases in Nochime. In EffectiveTags, Q20 and Q30 represent the proportions of bases with quality values exceeding 20 (sequencing error rate below 1%) and 30 (sequencing error rate below 0.1%), respectively. Effective (%) represents the ratio of the number of Nochime to the number of rawPE.**
